# Supplementary material for: A systematic method for surveying data visualizations and a resulting genomic epidemiology visualization typology: GEViT
Source: Bioinformatics. 2018 Sep 26;35(10):1668–76. doi: 10.1093/bioinformatics/bty832 (PMC6513170; doi:10.1093/bioinformatics/bty832)
Supplement: bty832_Supp [file bty832_supp.zip › bty832-Suppl_data/bty832_SupplementaryMaterials.pdf]

Supplemental Material for

A method for systematically surveying data visualizations in infectious disease genomic epidemiology

Anamaria Crisan, Jennifer Gardy, and Tamara Munzner

Contents

1. Supplemental Methods for Visualization Analysis (including references)
2. Supplemental Figures S1 to S3
3. Supplemental Table S1 to S4 Captions

A reminder that analysis notebooks are also available at:
`https://github.com/amcrisan/GEViTAnalysisRelease`

Supplemental Methods for Visualization Analysis

We applied qualitative analysis techniques in order to consistently describe and compare aspects of our corpus of literature-derived data visualizations. We used a Grounded Theory approach, which refers to a general set of techniques used by qualitative researchers to inductively analyze and construct a theory about some phenomenon that is "grounded" in data (Jacko, 2012). Grounded Theory is conceptually similar to unsupervised analysis methods used in quantitative research (Muller *et al.*, 2016), since both approaches rely on emergent pattern matching that is found within human-curated and labelled data rather than applying a specific hypothesis or theory; in qualitative methods, the human resolves the relevant patterns, in quantitative methods, the algorithm does. Qualitative research approaches are useful when trying to explore some data without any pre-conceived notions of what the outcomes should be.

The core foundation of Grounded Theory Methods (GTM) rests upon different approaches for assigning descriptive codes to data, typically chunks of text, that become the basis for further

analysis (Charmaz, 2006). Two widely used approaches are open and axial coding. In open coding, text is read multiple times to identify emergent themes – these are captured as codes. In axial coding, a researcher develops hierarchical relationships between codes. Codes are subjectively assigned to data and refined over multiple rounds of data interrogation until a final set of descriptive codes are agreed upon. Notions of validity and generalizability within qualitative research are different than within quantitative research, but internal validity is a recognized concept within qualitative research and there exist agreed-upon conventions to assess this validity (see (Maxwell, 2013), Chapter 6), which we have employed here.

GTM is used in the field of information visualization (infovis), though we note that the application of GTM is different between the social sciences and human-computer interaction (HCI). HCI and infovis researchers frequently apply GTM to text (Furniss *et al.*, 2011), video, and image data (Carpendale, 2008), whereas social scientists tend to primarily use interview text, although some examples of image analysis with social sciences exist (Liebenberg *et al.*, 2012). Our application of GTM, and especially open and axial coding, is drawn from the HCI and infovis research traditions, and we build upon established terminology and ideas from Munzner's Visualization Analysis and Design (Munzner, 2014). As our team comprises primarily quantitative researchers, we apply a specific interrogative lens to the way we use GTM. There exists a fascinating and broader discussion about mixed methods approaches that best combine qualitative and quantitative research methods (Creswell and Piano, 2007), which is beyond the application of this work but that the reader should be aware of.

Supplemental Methods References

Carpendale,S. (2008) Evaluating Information Visualizations. In, *Information Visualization*. Springer Berlin Heidelberg, pp. 19–45.

Charmaz,K. (2006) Constructing grounded theory: a practical guide through qualitative analysis Sage, London.

Creswell,J.W. and Piano,V.L. (2007) Designing and Conducting Mixed Methods Research. *Aust. N. Z. J. Public Health*, **31**, 388–388.

Furniss,D. *et al.* (2011) Confessions from a grounded theory PhD: Experiences and lesson learnt.

Jacko,J.A. (2012) Human-Computer Interaction Handbook: Fundamentals, Evolving Technologies, and Emerging Applications, Third Edition 3rd ed. CRC Press, Boca Raton, FL, USA.

Liebenberg,L. *et al.* (2012) Analysing image-based data using grounded theory: the Negotiating Resilience Project. *Vis. Stud.*, **27**, 59–74.

Maxwell,J.A. (2013) Qualitative Research Design: An Interactive Approach.

Muller,M. *et al.* (2016) Machine Learning and Grounded Theory Method: Convergence, Divergence, and Combination. *Proc. Gr.*, 0–6.

Munzner,T. (2014) Visualization Analysis and Design 1st ed. CRC Press, Boca Raton, FL, USA

Supplemental Figures

Figure S1A Literature Mining Methods.

Approach	Literature Search	Data Clean-up	Unsupervised Clustering	Linking to *a priori* Topics	Sampling
Data	Pubmed Central *Titles & Abstracts*	Document corpus	Tidytext corpus, Document term matrix	Tidytext corpus Document corpus	Document corpus
Methods	Query Pubmed through R	Extract 1-gram, Remove stop words, Remove numbers, remove common words, Calculate td_idf metric	rTSNE, HBSCAN (search for optimal hbscan params)<br><br>Name clusters by two most common names	Manual annotations	Sample per topic (per pathogen, see results)<br><br>Manually assess appropriateness, re-sample for rejected
Packages	`risemed`, `parseJSON`	`tidytext, snowballC, dplyr, Stringr`	`rTSNE, hdbscan`	-	-
Output	Document corpus	Tidytext corpus, Document term matrix	add cluster to document corpus<br><br>[a result]	add cross-cutting topic to document corpus<br>[a result]	Sampled document corpus<br>Spreadsheet keep/reject (reason)

Figure S1B Qualitative and Quantitative Visualization Analysis Methods.

Approach	Figure Extraction (including captions)	Axial Coding	Gallery Development	Quantitative Analysis
Data	Sampled Document Corpus<br>*+ some manual additions*	Figure (and table) corpus	Sampled Document Corpus<br>Figure & Tables<br>Code set	Sampled Document Corpus<br><br>Annotated Figures & Tables
Methods	Manually extract figures & some tables from PDF<br>Optical character recognition for figure captions	Manual, lots of group discussion and iterative refinement	Prototype development	Univariate & Bivariate Descriptive Statistics
Packages	`tesseract`	-	`shiny`	`dplyr;ggplot`
Output	Figures & some tables with captions as text	Code set for: basic chart types, chart combinations, and chart annotations [a result]	Annotated Figures & Tables<br>Browseable gallery<br>[results]	Descriptive Statistics<br><br>[a result]

Figure S2 *A priori* concepts distributed among pathogens (a) and the number of bigrams assigned to each concept (b).

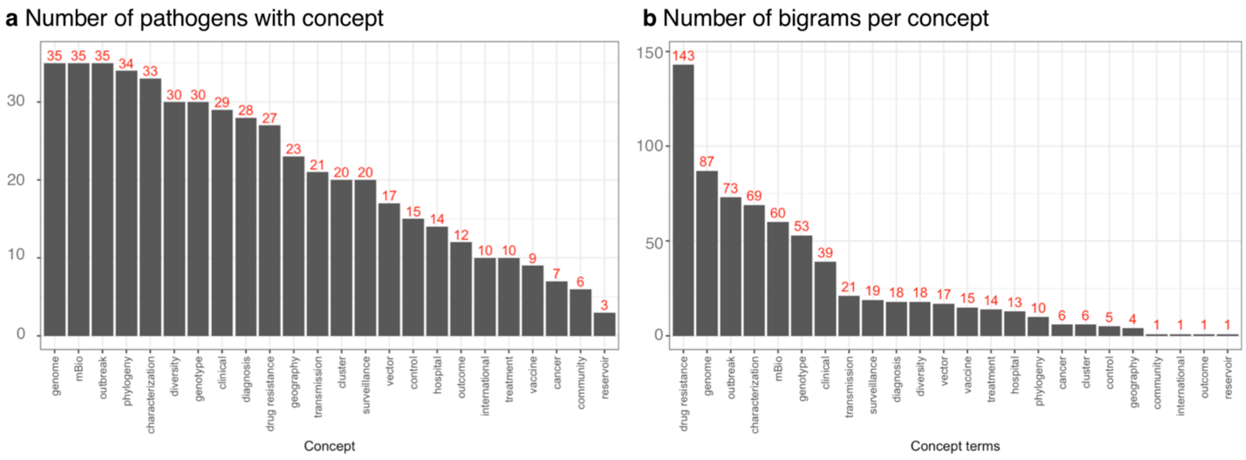

Figure S3 Distribution of chart types across articles (a) and the co-occurrence of chart types with figures (b)

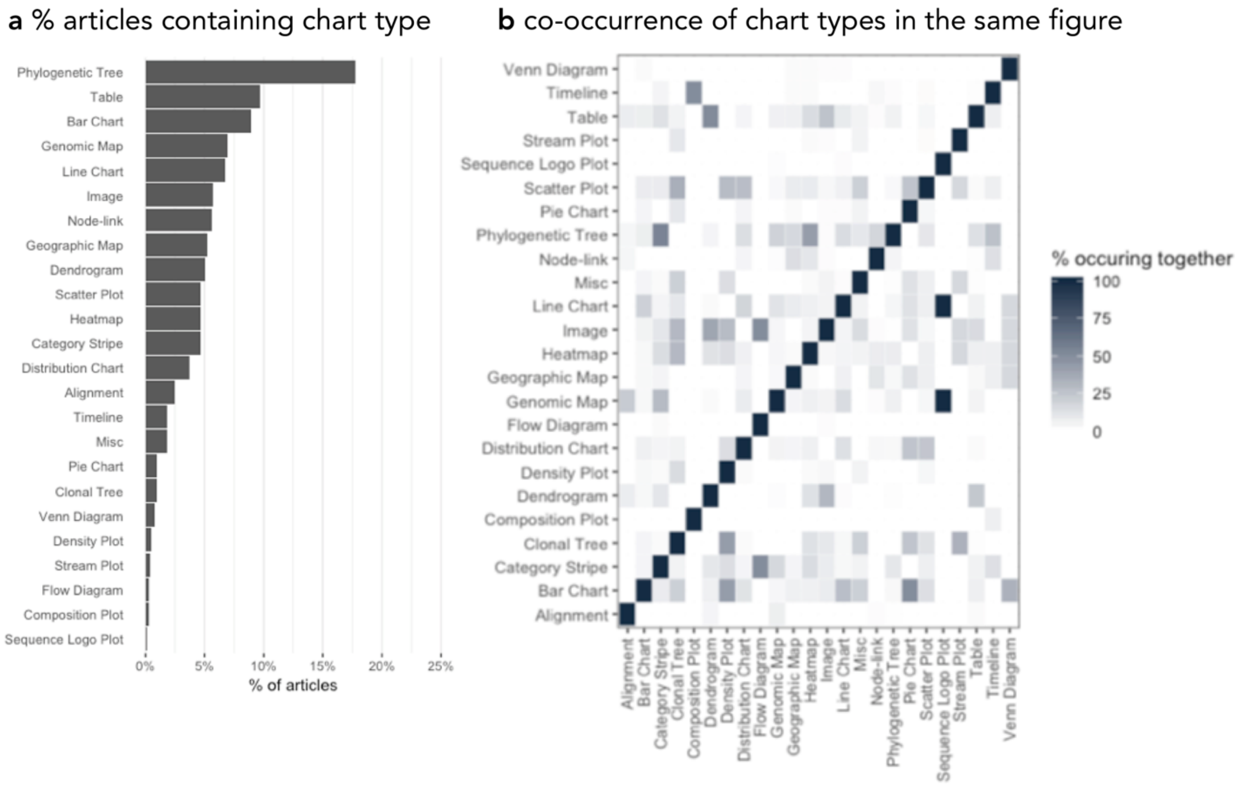

Supplemental Table Captions

Table S1 External list of pathogens. A list of human pathogens and their associated disease taken from Wikipedia (`https://en.wikipedia.org/wiki/List_of_infectious_diseases`) and used to validate the topic clustering by assessing whether the pathogen strings occur in clusters with the same name. Both the disease and the source of the disease were checked for a match within each document.

Table S2 Mapping of bigrams to concepts.

Table S3 Master list of sampled articles.

Table S4 Final Set of Pathogens and Pathogen Clusters
